# First in human phase 1 study of DT2216, a selective BCL-xL degrader, in patients with relapsed/refractory solid malignancies

**DOI:** 10.1186/s13045-025-01753-8

**Published:** 2025-11-12

**Authors:** Daruka Mahadevan, Minal Barve, Devalingam Mahalingam, Jay Parekh, Michael Kurman, James Strauss, Larry Tremaine, Robert Hromas, Joshua Sills, John McCulloch, John Harkey, Stacy Suberg, Lisa Zimmerman, Guangrong Zheng, Daohong Zhou

**Affiliations:** 1 Mays Cancer Center, San Antonio, TX USA; 2 Sarah Cannon Cancer Research, Dallas, TX USA; 3 Robert H. Lurie Comprehensive Cancer Center, Chicago, IL USA; 4 Dialectic Therapeutics, Dallas, TX USA; 5 University of Florida, Gainesville, FL USA

## Abstract

**Background:**

Small molecule inhibition of BCL-XL with navitoclax resulted in on-target dose-limiting thrombocytopenia. DT2216 was more effective than navitoclax and reduced platelet toxicity in preclinical models by selectively degrading BCL-XL via the VHL E3 ligase, which is minimally expressed in platelets.

**Methods:**

A dose escalation study using a 3 + 3 design with doses ranging from 0.04 to 0.4 mg/kg IV twice weekly (BIW) was performed. Eligible subjects had solid tumors of any histology that had progressed on standard treatment and had measurable tumor by RECIST v1.1. Tumor assessment was performed at 8-week intervals. BCL-XL levels were measured in peripheral leukocytes by western blotting.

**Results:**

Twenty patients were enrolled, with a median age of 60.5 year; 60% were female. Only one dose-limiting toxicity was observed, grade 4 thrombocytopenia that resolved within 48 h. Stable disease, observed in 20% of the patients. The lowest platelet count in the first cycle ranged from 24,000 to 297,000. In all cases, the platelet count recovered to > 50,000 within 4 days and > 75,000 within 1 week. There were no episodes of bleeding or treatment emergent adverse events leading to death. The median overall survival was 7.9 months. The plasma AUC of DT2216 was dose proportional with no dose accumulation. Patients receiving 0.4 mg/kg DT2216 demonstrated rapid and sustained degradation of BCL-XL.

**Conclusions:**

Based on the rapid recovery of transient thrombocytopenia that occurred only in the first cycle and the degradation of BCL-XL in peripheral leukocytes, the RP2D of DT2216 is 0.4 mg/kg IV BIW. (NCT04886622)

## Introduction

The evasion of apoptosis is a key mechanism that promotes the progression of cancer and resistance to anticancer therapies. This is partly attributable to the overexpression of anti-apoptotic proteins of the BCL-2 family, including BCL-2, BCL-XL, and MCL-1 [1]. However, the development of specific inhibitors of these proteins has been challenging because of insufficient activity, excessive toxicity, or both. Venetoclax, a BCL-2 inhibitor, is the only inhibitor of this family of anti–apoptotic proteins approved for the treatment of chronic lymphocytic leukemia (CLL), small lymphocytic lymphoma, and acute myeloid leukemia [2]. However, its use in cancer is limited because BCL-XL is a primary survival factor for most cancer cells and plays a unique role in the general resistance to cytotoxic agents [3]. A safe and effective BCL-XL target would be particularly useful as it would broaden the applicability of apoptosis inhibitors to other malignancies. However, mechanism-related toxicities, particularly thrombocytopenia, have presented drawbacks in the development of BCL-XL inhibitors [4].

DT2216 is a novel, rationally designed small-molecule degrader of BCL-XL protein, which functions as a molecule called proteolysis targeting chimeras (PROTACs) [5]. PROTACs are bivalent small molecules containing a pharmacophore that recognizes a target protein linked to a second pharmacophore that binds to a specific E3 ubiquitin ligase. DT2216 is the first drug candidate in a new class of bivalent small molecules targeting the BCL-2 family of proteins and is named Antiapoptotic Protein Targeted Degradation (APTaD™). As a bifunctional APTaD™, DT2216 binds to BCL-XL with one arm and the von Hippel–Lindau protein (VHL) E3 ligase with the other arm, which leads to BCL-XL ubiquitination and subsequent degradation of BCL-XL by the ubiquitin-proteasome system, which is a major pathway for intracellular protein degradation. Because human platelets express minimal levels of VHL, DT2216 is expected to be less toxic to platelets than other BCL-XL inhibitors, such as navitoclax (also known as ABT-263).

In preclinical pharmacological testing, DT2216 was shown to degrade BCL-XL in vitro, and this effect was rapid and long-lasting. In addition, DT2216 demonstrated in vitro anti-cancer activity against a broad range of tumor cells at low EC50 values. DT2216 demonstrated dose-dependent antitumor activity in vivo as a monotherapy for cutaneous T-cell lymphoma (CTCL) and demonstrated synergy in combination with various conventional chemotherapeutics and targeted drugs against a variety of hematological malignancies and solid tumors. This first-in-human phase 1 study was designed to determine RP2D, investigate the safety, tolerability, pharmacokinetics (PK), pharmacodynamics (PD) and efficacy of DT2216 in patients with advanced solid tumors.

## Methods

### Clinical study design

NCT04886622 is a phase 1, first-in-human, multicenter, open-label, dose escalation, and expansion study of DT2216, designed and sponsored by Dialectic Therapeutics, Inc. Data for the dose escalation phase are presented here. For the dose escalation portion of the study, the first patient was enrolled on 9–21-2021, and the last patient was enrolled on 8-8-2023.

The primary objective of the escalation phase was to determine the recommended phase 2 dose (RP2D) for DT2216. The secondary objectives were to characterize the adverse event (AE) profile, determine the pharmacokinetic profile (PK)/pharmacodynamic profile (PD) and describe preliminary evidence of efficacy using objective response rate (ORR), disease control rate (DCR), and progression-free survival (PFS) based on Response Evaluation Criteria in Solid Tumors (RECIST) v1.1. Exploratory objectives were to characterize the baseline and on-treatment expression of BCL-XL and VHL on blood lymphocytes, as well as other circulating biomarkers. Exploratory objectives included characterizing the baseline and on-treatment changes in platelet count and correlating them with DT2216 drug levels.

Eligible patients included those with advanced histologically or cytologically confirmed solid tumors that exhausted all treatment options or had a contraindication to approved therapies or generally recognized standard-of-care measures for their cancer after having progressed or being intolerant to the last treatment as determined by the investigators. Patients were aged ≥ 18 years and had an Eastern Cooperative Oncology Group (ECOG) performance status of 0 or 1. Patients had evaluable or measurable disease according to RECIST v1.1. Patients had to have an adequate marrow reserve with a baseline platelet count *≥* 100,000 and adequate liver and renal function. Key exclusion criteria included prior BCL-XL directed therapy, history of a major surgical procedure, growth factor use, blood products, participation in another investigational agent study within 1 month prior to commencing the study drug, and known active brain metastases/carcinomatous meningitis. The full eligibility criteria are available in the protocol (Supplementary Information).

Dose escalation consisted of the administration of DT2216 intravenously twice weekly at several different doses outlined in protocol attached. The starting dose was 0.04 mg/kg, established based on pre-clinical toxicity studies. Safety was evaluated using CTCAE version 5.0. The initial three dose levels enrolled one patient, following which for dose level 4 and 5, a 3 + 3 design was utilized following a 100% incremental dose increase for dose levels 2 to 5 until the first dose limiting toxicity (DLT) in cycle 1 was observed. Thereafter, a modified Fibonacci sequence was used to define dose escalation increments until the maximum tolerated dose (MTD) was reached as described in protocol. If no dose-limiting toxicity (DLT) was observed during the DLT monitoring period of first 28 days, the next patient was enrolled at the next higher dose level. Dose-limiting toxicities (DLTs) during the first treatment cycle were defined as any Grade 5 toxicity; Grade 4 neutropenia lasting > 7 days; febrile neutropenia; Grade 4 thrombocytopenia or Grade 3 thrombocytopenia with clinically significant hemorrhage or lasting ≥ 7 days; and Grade ≥ 3 non-hematologic toxicities, excluding nausea, vomiting, or diarrhea lasting ≤ 3 days or transient laboratory abnormalities resolving within 72 h. Hepatotoxicity meeting Hy’s law criteria was also considered a DLT. Transient Grade 3–4 electrolyte abnormalities resolving to ≤ Grade 2 within 72 h were not considered DLTs.

Patients were allowed a maximum of two intra-patient dose escalations. Patients received DT2216 by intravenous infusion on days 1 and 4 weekly for at least 4 weeks, with each cycle consisting of a 28-day cycle for a maximum duration of 1 year until disease progression, unacceptable toxicity, or other withdrawal criteria, as listed in the protocol. The extent of disease was evaluated using imaging studies and disease-appropriate tumor markers every 8 weeks from day 1 of the first cycle. RP2D was established based on MTD as well as other relevant data, including clinical signals of activity, PK, and PD data.

Premedication prior to each infusion was at the discretion of the investigator or according to institutional guidelines. Patient assessment and follow-up procedures can be found in the schedule of assessments in this study. The clinical response was evaluated using RECIST v1.1 per protocol. Blood samples were collected for PK analysis before and after post-infusion at defined intervals, along with platelet counts. Exploratory biomarkers include (but are not limited to) the assessment of BCL-XL and VHL levels in peripheral white blood cells (WBC).

All relevant ethical regulations were followed during this study. The methods were performed in accordance with relevant guidelines and regulations and approved by the Food and Drug Administration (FDA). Written informed consent was obtained from all study participants. The Institutional Review Boards (IRBs) of all participating institutions approved the study protocol. The institutions participating in the study were Northwestern University Medical School, Chicago, IL; Mary Crowley Cancer Research, Dallas, TX; and Mays Cancer Center, UT Health San Antonio, TX.

We used the CONSORT checklist when writing our report.

#### Inclusion criteria

The full eligibility criteria are available in the protocol (supplementary information). Subjects were eligible to participate in the study if they met the following criteria: (1) Adults aged 18 years or older on the day of signing the informed consent form. (2) Written informed consent was provided for the trial, including the willingness to comply with all study-related requirements. (3) Histologically or cytologically confirmed solid tumors. (4) Evidence of disease progression or inadequate response to the last regimen was assessed by an investigator. (5) Has exhausted standard of care options or had contraindications. (6) The presence of measurable disease as assessed by the Investigator per Response Evaluation Criteria in Solid Tumors (RECIST) v1.1. (7) An Eastern Cooperative Oncology Group (ECOG) performance status of 0 or 1. (8) Has adequate organ functions as defined by parameters outlined in protocol.

## Exclusion criteria

Subjects were excluded from participation if they met any of the following criteria:1. received prior BCL-XL directed therapy. 2. Administration of any blood product within 30 days preceding the planned administration of study therapy. 3. History of clinically significant bleeding and/or bleeding predisposition. 4. Significant liver insufficiency was defined as Child-Pugh class B or C. 5. Concurrent administration of medications or foods that are strong inhibitors or inducers of cytochrome P4503A (CYP3A). Strong CYP3A inhibitors and inducers should be discontinued at least two weeks prior to the first dose of DT2216. 6. Known active central nervous system (CNS) metastases/and or carcinomatous meningitis. A complete list of detailed exclusion criteria are available in protocol attached.

### Statistical analysis

Descriptive statistics were used to display dose escalation data and results. Continuous variables, including baseline characteristics, will be summarized by reporting the number of observations, mean, standard deviation, median, minimum, and maximum values. Categorical/discrete variables will be summarized using frequency tables that show the number and percentage of subjects within a category. Time-to-event analysis was performed using the Kaplan–Meier method. The overall response rate (ORR) and its 2-sided 95% confidence interval (CI) were calculated using the Clopper-Pearson method.

## Results

### Clinical patient population, treatment, and disposition

**Fig. 1:**
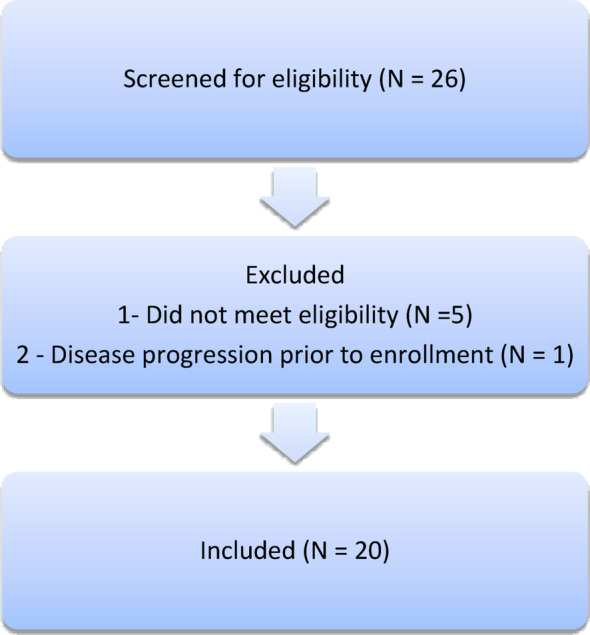
Consort flow diagram of patients enrolled in DT2216 phase 1 trial

Twenty patients who received at least 1 dose of DT2216 were evaluated after initial screening (Fig. 1). Patient demographics and disease characteristics according to dose cohort are shown in Table 1. The median age was 60.5 years (range 30–80) and 100% of patients had an ECOG performance status of0 or 1. A variety of tumor types were enrolled, as shown in Table 1.

**Table 1:** Demographics

Baseline characteristics	Cohort (dose mg/kg)						
	1 (0.04)	2 (0.08)	3 (0.12)	4 (0.24)	5 (0.32)	6 (0.40)	Total
**N**	1	1	1	7	6	4	20
**Median Age** (range)	54 (54)	65 (65)	50 (50)	68 (48–76)	47 (30–80)	71.5 (52–74)	60.5 (30–80)
**M/F**	0/1	0/1	0/1	3/4	3/3	2/2	8/12
**Race**							
**Caucasian**	1	1	1	5	3	3	14
**African American**	0	0	0	2	1	0	3
**Asian**	0	0	0	0	1	1	2
**Unknown**	0	0	0	0	0	1	1
**ECOG**							
**0**	0	0	1	2	3	3	9
**1**	1	1	0	5	3	1	11
**Median No. Prior Systemic Therapies**	7	5	5	3	3	4	4
**Tumor Type**							
Colorectal							5
Pancreas							4
Ovarian							2
NSCLC							2
Thymus							2
*Breast*							1
*Liver*							1
*SCLC*							1
*Other*							2

**Table 2:** Patient disposition

Event	Cohort (dose mg/kg)						
	1 (0.04)	2 (0.08)	3 (0.12)	4 (0.24)	5 (0.32)	6 (0.40)	Total
**Subjects treated**	1	1	1	7	6	4	20
**Subjects discontinuing treatment**	1	1	1	7	6	4	20
**Reason for treatment discontinuation**							
Adverse event	0	0	0	0	0	0	0
Withdrawal by subject	0	0	0	1	0	0	1
Clinical progression of neoplastic disease	1	0	0	2	1	0	4
Objective progression of neoplastic disease	0	1	1	4	5	4	15

**Fig. 2:**
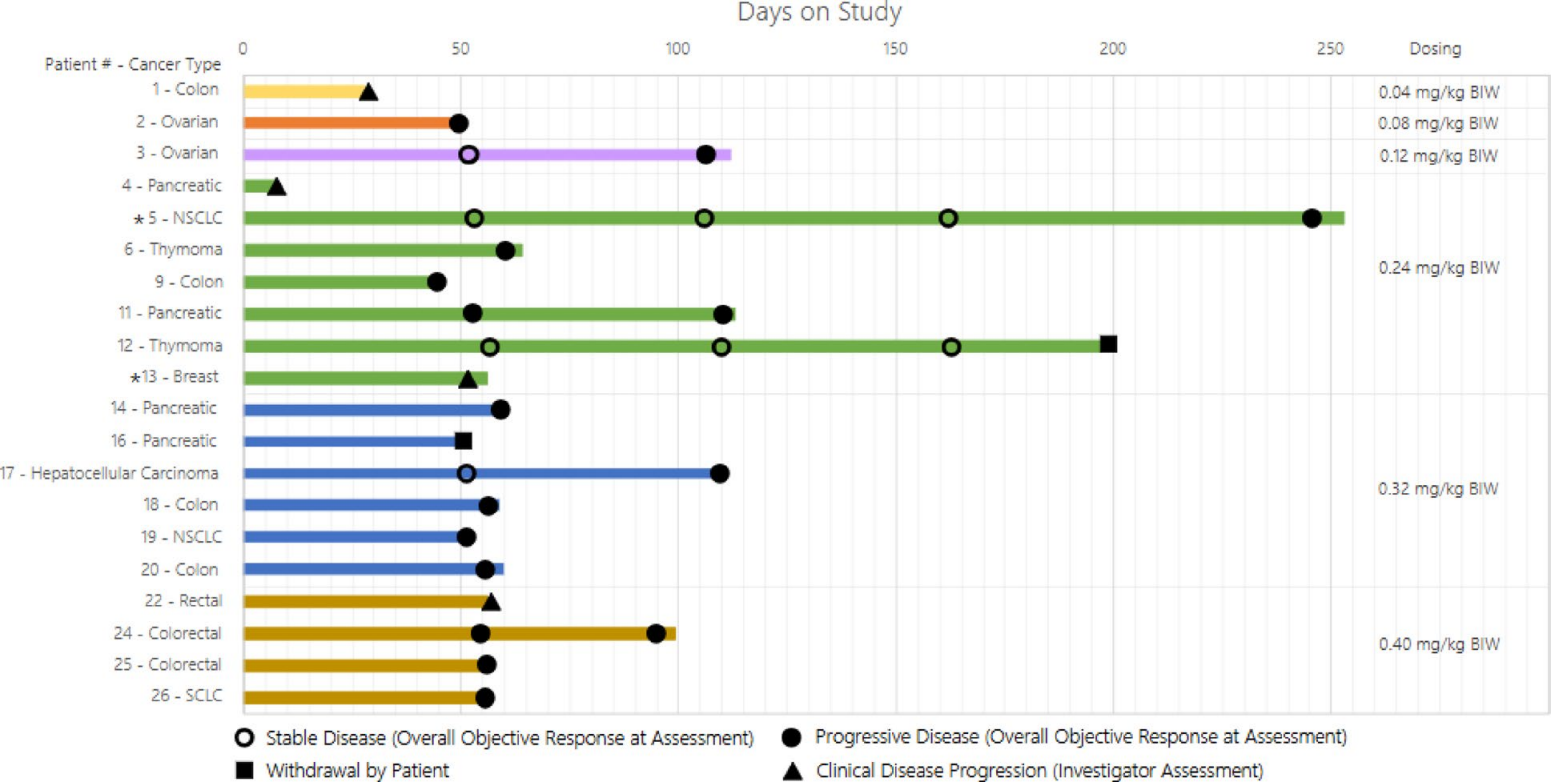
Swimmer plot of individual patients on the trial. Each horizontal bar represents individual patient with the tumor type as listed on the vertical left axis and their duration on study with radiological response per RECIST v1.1. The bars are color coded according to the dose cohort. Note: Patients 7, 10, 15, 21 & 23 did not pass screening. Patient 8 experienced disease progression prior to enrollment. * Patients 5 and 13 dose reduced from 0.24 mg/kg BIW to 0.12 mg/kg BIW at beginning of Cycle 3 and Cycle 2 of treatment, respectively. End of treatment objective response assessment not performed for patients 1, 4, 12, 16 and 22

The patient population was heavily pre-treated; all patients received ≥ 3 prior treatment regimens for advanced/metastatic disease. All 20 patients received one dose of DT2216, and 19 completed the DLT assessment period (C1D28), as one patient chose to proceed with hospice care after receiving an initial dose due to disease-related clinical decline. While none of the patients had dose escalation, dose interruption occurred in 8 patients due to transient thrombocytopenia, and 2 patients had dose reduction due to repeated thrombocytopenia that resolved. The highest number of treatment cycles given to a patient was 9, and the median duration of treatment was 57 days (range, 7–253) (Fig. 2). 15/20(75%) patients discontinued treatment because of objective disease progression. Reasons for treatment discontinuation are listed in Table 2.

**Fig. 3:**
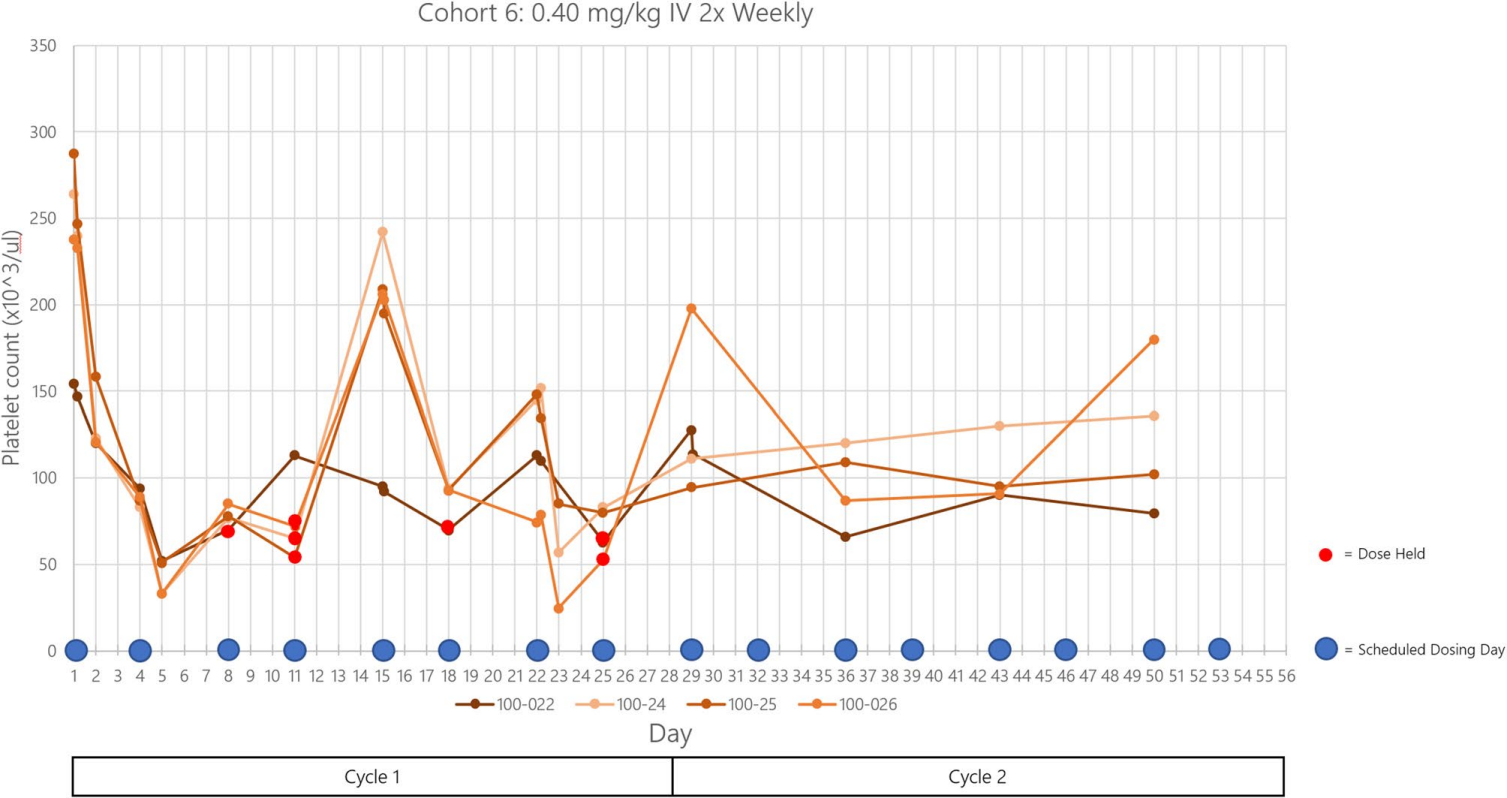
Trend of platelet count of cohort 6 patients at DT2216 dose 0.40 mg/kg IV BIW. Vertical axis represents the platelet count (x10^3^/microliter) and horizontal axis represents the days on study. Rebound of platelets post-dosing demonstrates the regenerative nature of bone marrow and that megakaryocytes are not harmed. *DLT*,* PK and RP2D*

The incidence of ≥ grade 3 AEs suspected to be related to the study drug was very low 4/20 (20%). Sixteen patients (80%) experienced at least one related treatment-emergent adverse event (TEAE). A TEAE is defined as any event that occurs on or after the first dose of the study drug administration or any pre-existing event that worsened in severity after dosing. There were no major bleeding events or treatment related deaths. Thrombocytopenia was the most common adverse event but none of the patients required platelet transfusions during the study. 8/20 patients had dose interruption due to thrombocytopenia with median duration of dose interruption of 5 days. 1 patient had dose reduction due to thrombocytopenia. The most frequently related TEAEs are shown in Table 3: thrombocytopenia (9 patients, 45%), fatigue (2 patients, 10%), and infusion-related reaction (2 patients, 10%). The lowest platelet count observed during the study was 24,000/µL. (Fig. 3).

**Table 3:** Adverse Events

Event	Number of subjects (%)	Grade > 3
Thrombocytopenia	9 (45)	1
Fatigue	2 (10)	0
Infusion related reaction	2 (10)	0
Alanine aminotransferase increased	1 (5)	0
Blood bilirubin increased	1 (5)	0
Lipase increased	1 (5)	0
Temperature intolerance	1 (5)	0
Contusion	1 (5)	0
Decreased appetite	1 (5)	0
Hematuria	1 (5)	0

**Fig. 4:**
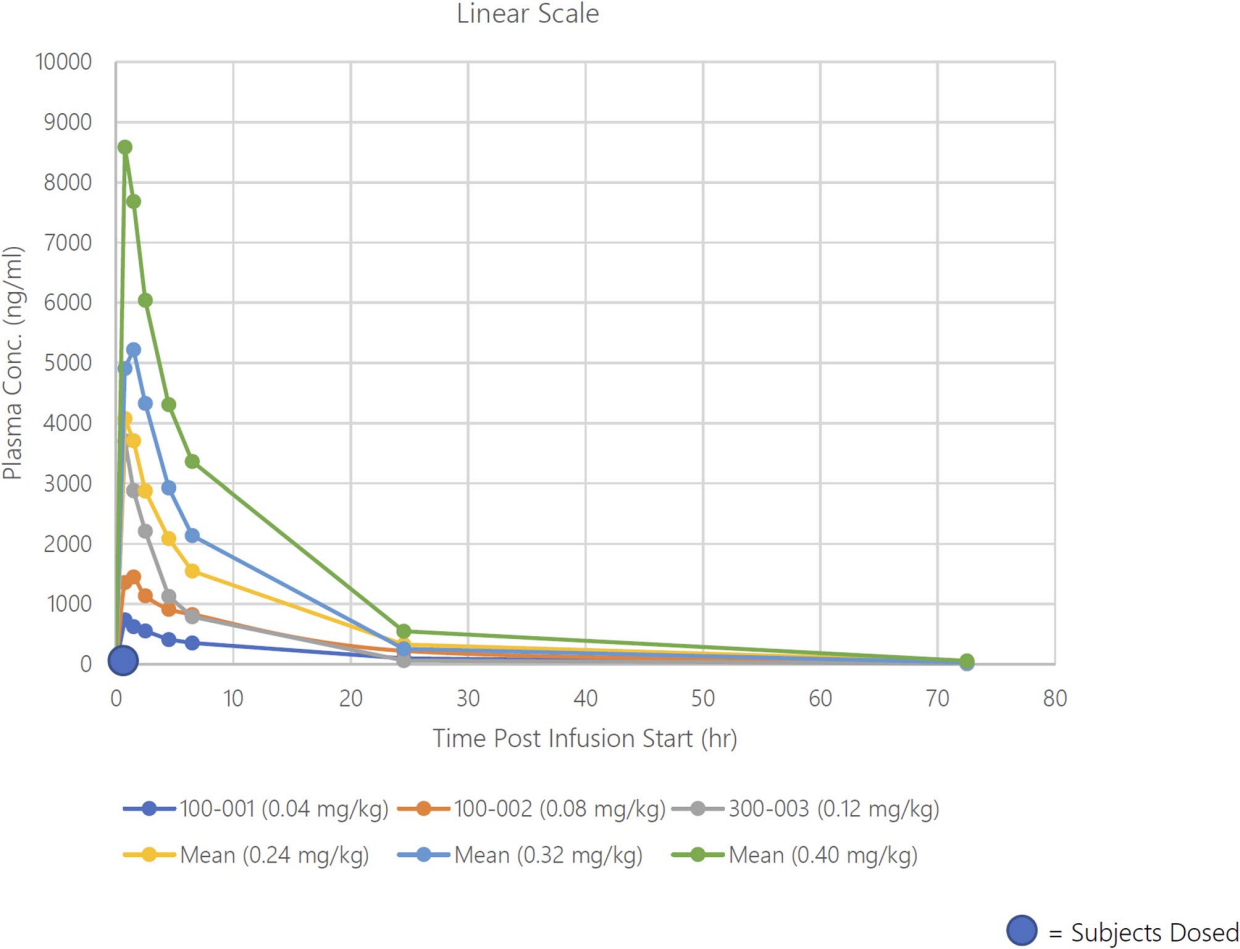
Plasma concentration of DT2216 on vertical axis(nanogram/milliliter) and time in hours post infusion start (30-minute infusion duration) on horizontal axis. The PK profile is consistent with a dose proportional increase in DT2216 exposure

**Fig. 5:**
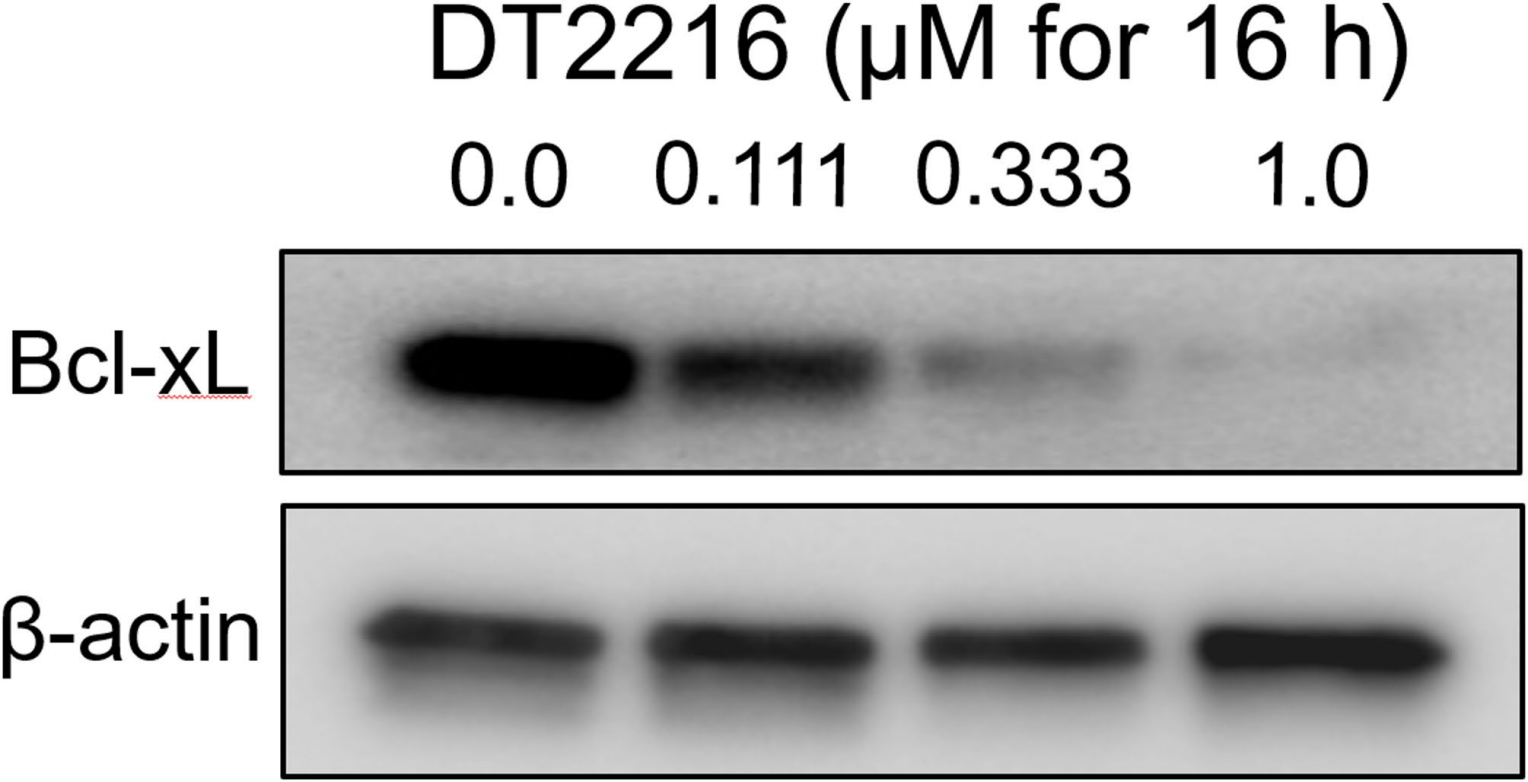
Levels of BCL-XL in WBCs from a normal human donor. They were determined after incubation with increasing concentrations of DT2216 as depicted in the figure for approximately 16 hours​. We observe a dose dependent degradation. BCL-XL expression after incubation with DT2216 in comparison to beta actin which acts as control in the figure

**Fig. 6:**
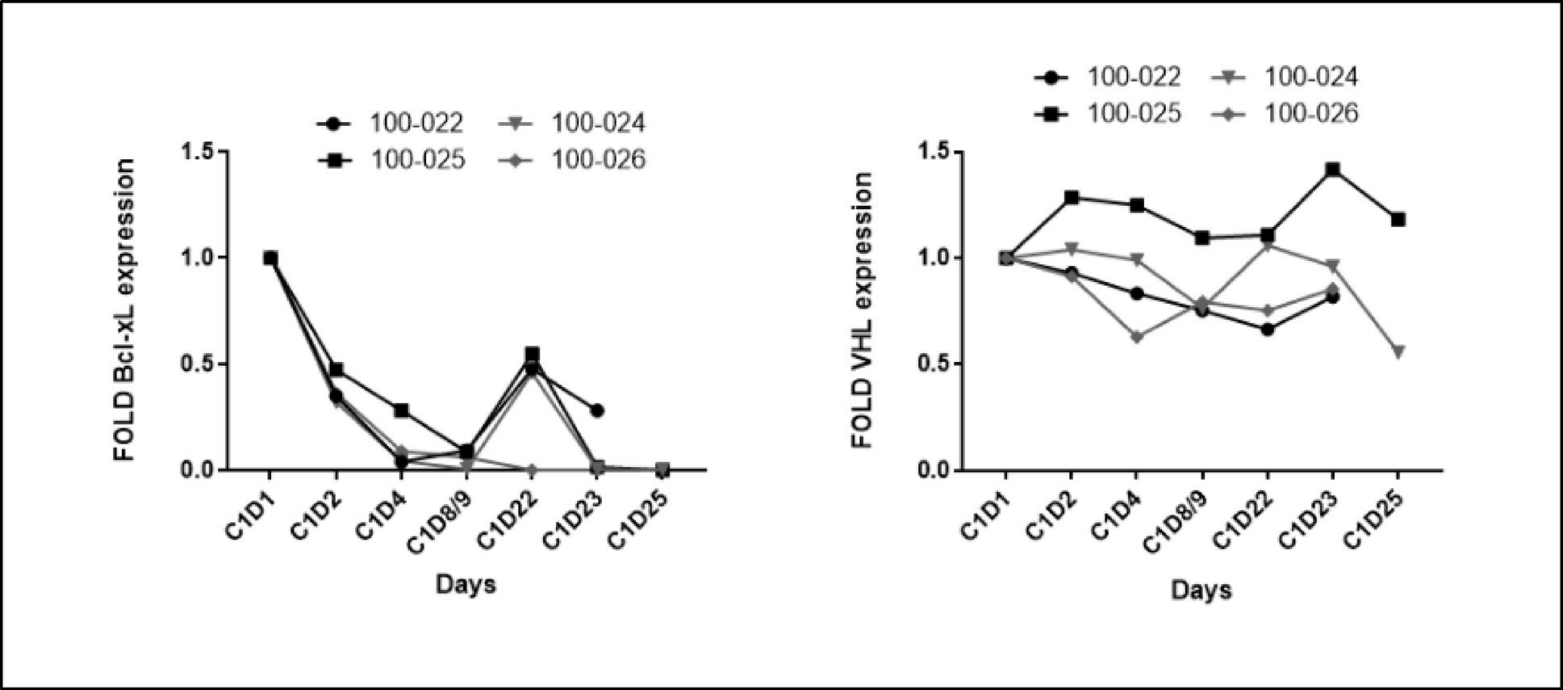
Changes in BCL-XL and VHL expression after infusion of DT2216 at 0.4 mg/kg in 4 patients. Left panel shows changes in fold BCL XL expression after C1D1. Expression of BCL XL decreases rapidly after infusion of DT2216 and is persistent. Right panel depicts fold changes in VHL expression in the same patients after infusion of DT2216. VHL expression did not demonstrate a simultaneous drop in contrast to BCL XL

**Fig. 7:**
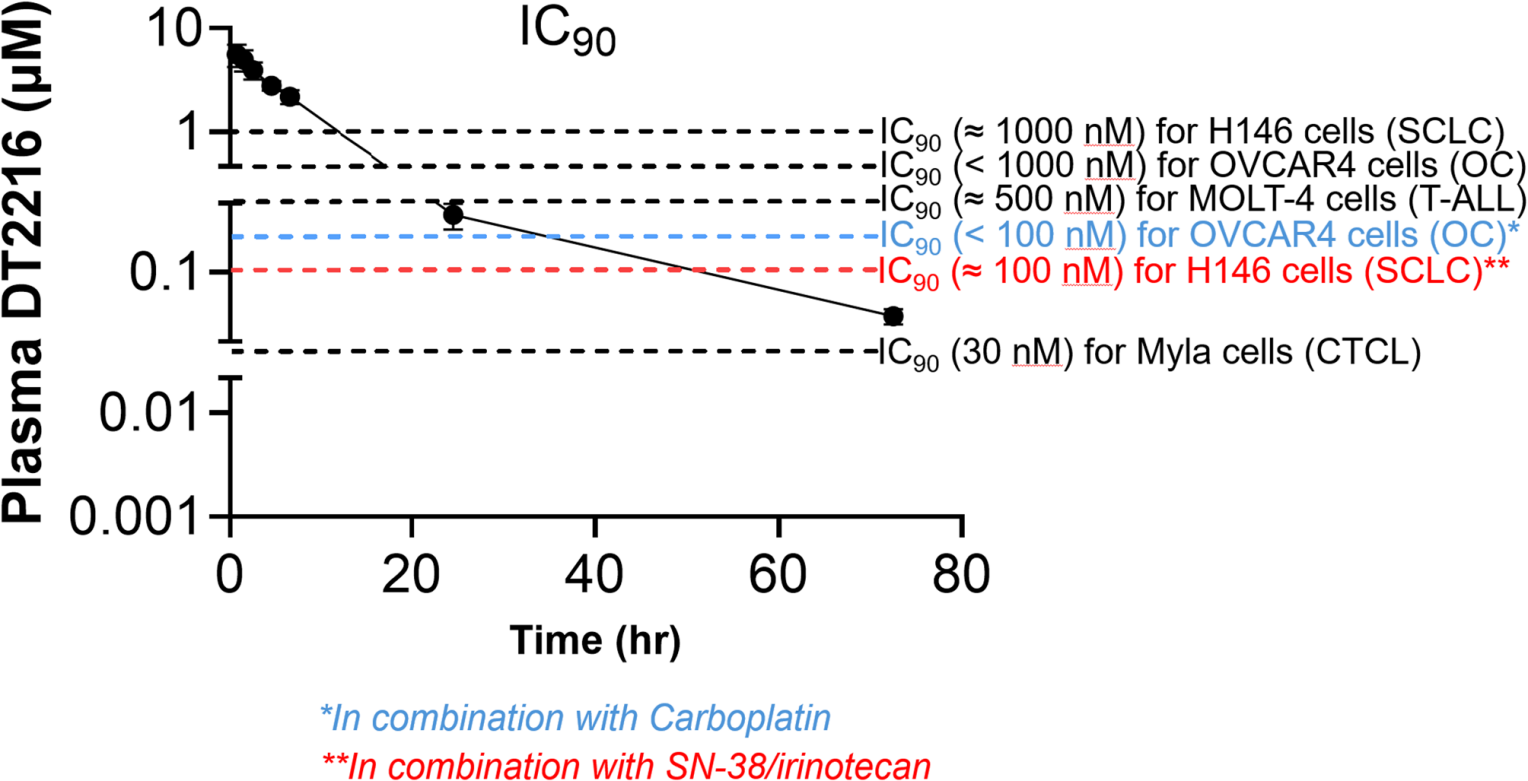
Mean Plasma concentration of DT2216 in 4 patients infused with DT2216 at 0.4 mg/kg over time against in vitro inhibitory concentration 90%(IC_90_) for various cell lines. SCLC – small cell lung cancer; OC- ovarian carcinoma; T-ALL – T cell acute lymphoblastic leukemia, CTCL – Cutaneous T cell lymphoma

Throughout the course of the dose escalation trial, only one dose-limiting toxicity was observed, with grade 4 thrombocytopenia at a dose level of 0.32 mg/kg. Figure 4 shows the plasma concentration of DT2216, with time = 0 representing the start of a 30-minute intravenous infusion. The pharmacokinetic parameters of DT2216 were determined in patients following doses on Cycle 1 Day 1 and Cycle 1 Day 22 (Tables 4 and 5). Following IV infusion of DT2216, the Cmax and AUC values for DT2216 increased with increasing dose, with mean Cmax values ranging from 738 ng/mL to 8590 ng/mL in the dose range of 0.04 mg/kg 0.4 mg/kg. AUC0-∞ values ranged from 8650 to 72,300 ng · h/mL over the dose range of 0.04 mg/kg 0.4 mg/kg. DT2216 was eliminated from the plasma with a mean half-life ranging from 7.09 to 12.8 h over the dose range tested and was independent of dose. DT2216 clearance values ranging from 4.10 to 7.89 mL/min/kg were relatively constant over the dose range as well. The volume of distribution at steady state (Vss) was small (< 100 mL/kg) and consistent across the dose groups. Following repeated dosing, accumulation of DT2216 in plasma was not observed with twice weekly dosing, with accumulation ratio values ranging from 0.74 to 1.07 on Cycle 1 Day 22 (the fifth dose) across the dose groups. Based on pre-dose and 2-hour post infusion plasma DT2216 concentrations, steady state appeared to be reached by at least Cycle 1, Day 15, and to be maintained out to Cycle 9 in patients who were continuing the study. Figure5 shows that clinically relevant DT2216 plasma concentrations are capable of completely degrading BCL-XL in normal human WBCs. Figure 7 demonstrates the mean plasma concentration of DT2216 in micromolar in 4 patients infused with DT2216 at 0.4 mg/kg over time in hours against inhibitory concentration 90%(IC_90_) for various cell lines. Figure 6 shows the pharmacodynamic profiles of BCL-XL and VHL after C1D1 of DT2216. Based on these data, RP2D was chosen to be 0.4 mg/kg BIW.

**Table 4:** Mean (CV%) DT2216 plasma Pharmacokinetic parameters in patients from Cycle 1, day 1

PK parameters	DT2216 0.04 mg/kg (*N* = 1)	DT2216 0.08 mg/kg (*N* = 1)	DT2216 0.12 mg/kg (*N* = 1)	DT2216 0.24 mg/kg (*N* = 7)	DT2216 0.32 mg/kg (*N* = 6)	DT2216 0.4 mg/kg (*N* = 4)
T_max_, hour	0.75 (*N* = 1)	1.50 (*N* = 1)	0.75 (*N* = 1)	0.75 (0.75, 0.75) (*N* = 7)	0.75 (0.75, 1.50) (*N* = 6)	0.75 (0.75, 0.75) (*N* = 4)
C_max_, ng/mL	738 (*N* = 1)	1450 (*N* = 1)	3710 (*N* = 1)	4080 (12.0) (*N* = 7)	5710 (20.4) (*N* = 6)	8590 (27.9) (*N* = 4)
AUC_0−24_, ng*h/mL	5920 (*N* = 1)	13,400 (*N* = 1)	12,900 (*N* = 1)	24,500 (31.9) (*N* = 6)	33,300 (12.7) (*N* = 6)	53,000 (15.1) (*N* = 4)
AUC_last_^a^, ng*h/mL	8470 (*N* = 1)	19,100 (*N* = 1)	17,900 (*N* = 1)	37,600 (22.8) (*N* = 6)	43,200 (21.9) (*N* = 6)	72,300 (15.7) (*N* = 4)
AUC_0−∞_^b^, ng*h/mL	8650 (*N* = 1)	19,500 (*N* = 1)	18,000 (*N* = 1)	36,500 (31.4) (*N* = 7)	41,500 (16.7) (*N* = 5)	73,200 (15.6) (*N* = 4)
T½^b^, hour	12.8 (*N* = 1)	12.5 (*N* = 1)	8.92 (*N* = 1)	10.4 (29.4) (*N* = 7)	7.09 (35.4) (*N* = 5)	10.7 (7.70) (*N* = 4)
CL^b^, mL/min/kg	4.62 (*N* = 1)	4.10 (*N* = 1)	6.67 (*N* = 1)	7.32 (38.7) (*N* = 7)	7.89 (17.4) (*N* = 5)	5.57 (15.5) (*N* = 4)
Vd_ss_^b^, mL/kg	77.6 (*N* = 1)	70.0 (*N* = 1)	55.7 (*N* = 1)	83.9 (15.8) (*N* = 7)	68.3 (15.4) (*N* = 5)	70.9 (18.3) (*N* = 4)

AUClast = area under the curve from time zero to last measurable concentration; AUC0-∞=area under the curve from time zero to infinity; CL = clearance; Cmax = maximum plasma concentration; t1/2 = terminal elimination half-life; Tmax = time to Cmax; Vss = volume of distribution; CV = coefficient of variation. Tmax is presented as median (minimum, maximum). Tmax is measured from the start of the infusion. Preliminary PK parameters were calculated on intended and not actual sampling times. Plasma samples for Cycle 1 Day 1 were obtained pre-dose, and 0.25, 1, 2, 4-, 6-, 24- and 72-hour post-infusion for calculation of PK parameters

^a^2 patients on Cycle 1 Day 1 had samples collected only up to 24 h. The AUClast values for these two subjects are not included as AUClast is defined as area under the curve from time zero to last measurable concentration

^b^for 1 patient, the elimination phase for this subject could not be adequately estimated for half-life, CL, Vdss and AUC0-∞ could not be calculated

**Table 5:** Mean (CV%) DT2216 plasma Pharmacokinetic parameters in patients from cycle 1, day 22

PK parameters	DT2216 0.04 mg/kg (*N* = 1)	DT2216 0.08 mg/kg (*N* = 1)	DT2216 0.12 mg/kg (*N* = 2)	DT2216 0.24 mg/kg (*N* = 7)	DT2216 0.32 mg/kg (*N* = 6)	DT2216 0.4 mg/kg (*N* = 4)
T_max_, hour	2.50 (*N* = 1)	2.50 (*N* = 1)	2.50 (*N* = 1)	2.50 (2.50, 2.50) (*N* = 6)	2.50 (2.50, 2.50) (*N* = 6)	2.50 (2.50, 2.50) (*N* = 4)
C_max_, ng/mL	541 (*N* = 1)	962 (*N* = 1)	2190 (*N* = 1)	3090 (26.8) (*N* = 6)	4480 (10.0) (*N* = 6)	6270 (25.2) (*N* = 4)
AUC_0−24_, ng*h/mL	5350 (*N* = 1)	9840 (*N* = 1)	13,900 (*N* = 1)	26,300 (30.9) (*N* = 6)	32,700 (10.2) (*N* = 5)	53,700 (23.0) (*N* = 4)
AUC_last_^a^, ng*h/mL	5390 (*N* = 1)	9910 (*N* = 1)	13,900 (*N* = 1)	26,400 (30.9) (*N* = 6)	32,900 (10.3) (*N* = 5)	54,000 (23.0) (*N* = 4)
AUC_0−∞_, ng*h/mL	6230 (*N* = 1)	11,700 (*N* = 1)	14,500 (*N* = 1)	30,100 (35.2) (*N* = 6)	35,000 (11.0) (*N* = 5)	59,500 (23.1) (*N* = 4)
T½^a^, hour	8.18 (*N* = 1)	8.63 (*N* = 1)	5.24 (*N* = 1)	7.27 (33.5) (*N* = 6)	5.82 (9.5) (*N* = 5)	6.70 (14.8) (*N* = 4)
CL^a^, mL/h/kg	6.42 (*N* = 1)	6.84 (*N* = 1)	8.25 (*N* = 1)	8.99 (40.4) (*N* = 6)	9.23 (11.3) (*N* = 5)	7.01 (24.2) (*N* = 4)
Vd_ss_^a^, mL/kg	81.0 (*N* = 1)	91.0 (*N* = 1)	66.5 (*N* = 1)	93.9 (25.5) (*N* = 6)	83.7 (9.5) (*N* = 5)	72.9 (19.7) (*N* = 4)
ArA_UC0−24_	0.90 (*N* = 1)	0.74 (*N* = 1)	1.07 (*N* = 1)	1.08 (11.6) (*N* = 6)	1.02 (6.7) (*N* = 5)	1.01 (8.7) (*N* = 4)

ArAUC0-24 h = accumulation ratio; AUClast = area under the curve from time zero to last measurable concentration; AUC0-∞=area under the curve from time zero to infinity; CL = clearance; Cmax = maximum plasma concentration; CV = coefficient of variation; t1/2 = terminal elimination half-life; Tmax = time to Cmax; Vss = volume of distribution. Tmax is presented as median (minimum, maximum). Tmax is measured from the start of the infusion. Preliminary PK parameters were calculated on intended and not actual sampling times. Plasma samples for Cycle 1 Day 22 were obtained pre-dose and 2-, 4-, 6-, and 24-hour post-infusion for calculation of PK parameters

^a^One patient on Cycle 1 Day 22 had samples collected only up to 6 h, therefore half-life, CL, Vdss and AUClast, values for this subject were not calculated

## Efficacy

**Fig. 8:**
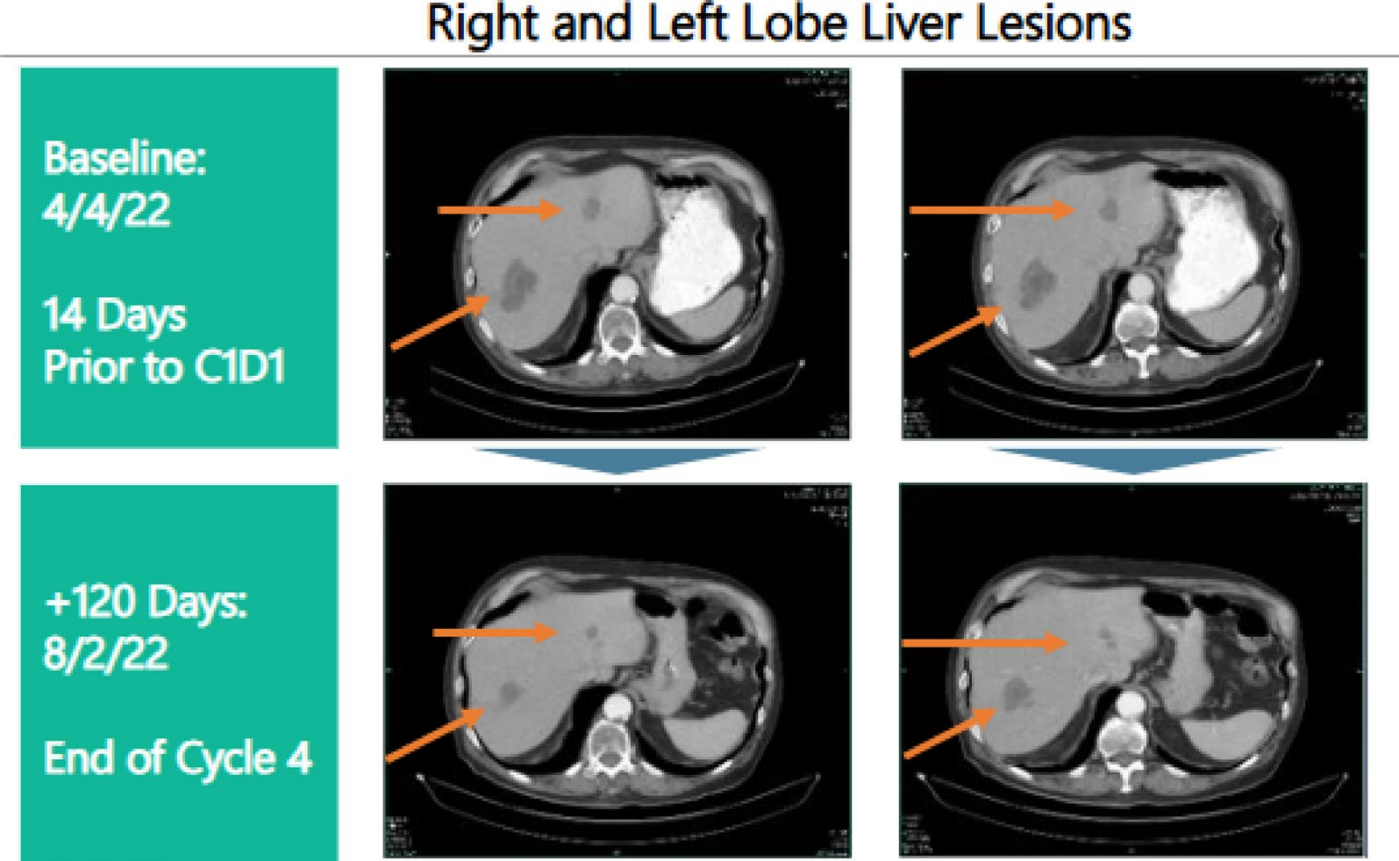
Target liver lesions in a 70-year-old male with non-small cell lung adenocarcinoma refractory to prior lines of treatment on dose level 0.24 mg/kg. Panel above shows target liver lesions at baseline 14 days prior to C1D1 infusion of DT2216. Panel below shows a decrease in size of target liver lesions after completion of C4 of DT2216

While efficacy was a secondary endpoint for evaluation in this trial, DT2216 did not demonstrate single-agent activity in patients with different advanced solid tumors, which was not unexpected because most cancer cells require a pro-apoptotic signal triggered by chemotherapy to respond to BCL-XL inhibition [5]. The best response per RECIST v1.1 criteria was stable disease which noted in 20% of patients with a median duration of response of 107 days. The median overall survival, as estimated by the Kaplan–Meier method was 7.9 months (95% CI 4.0 – NE). Figure 7 shows the response to target liver lesions at the end of C4 in comparison with baseline prior to starting DT2216 in a 70-year-old male with non-small cell lung adenocarcinoma refractory to three prior lines of treatment (Fig. 8).

## Discussion

Here, we report the first-in-human trial of DT2216, a BCL-XL PROTAC, in patients with advanced solid tumors that were refractory to multiple lines of prior systemic therapies. The primary aim of this study was to identify RP2D and assess the safety/tolerability, PK/PD, and anti-tumor activity of this first-in-class investigational agent, which targets an important anti-apoptotic pathway in human malignancies.

DT2216 treatment in this population was safe and well tolerated apart from the grade 4 thrombocytopenia. The major drug-related toxicity was asymptomatic thrombocytopenia, with no evidence of major bleeding and rapid recovery within 3–4 days. No other ≥ grade 3 adverse events were suspected to be related to the study drug were identified. None of the patients with thrombocytopenia required platelet transfusions and there were no major hemorrhagic events during the study. It was shown previously that platelets but not megakaryocytes and hematopoietic stem progenitor cells (HSPCs) are solely dependent on BCL-xL for survival because megakaryocytes and HSPCs also express MCL-1. BCL-xL inhibition or degradation can kill mature platelets but has no significant effect on megakaryopoiesis and hematopoiesis [6]. There were no treatment-related deaths as determined by the clinical principal investigators, and all patients completed the DLT assessment period except one patient who chose hospice care due to clinical decline unrelated to the study drug.

The pharmacokinetic (PK) profile showed a dose-dependent increase in exposure and maximum concentration with increasing dose levels. The maximum concentration of 8590ng/ml was observed at 0.4 a BIW dose. DT2216 has a small dose-independent volume of distribution and dose-independent clearance of approximately 7 ml/min/kg with a low coefficient of variation, favoring a stable pharmacokinetic profile. DT2216 was eliminated from plasma in a dose independent fashion with mean half-life ranging between 7 and 12 h irrespective of dose level and accumulation ratio between 0.7 and 1.01 even at the highest dose level of 0.4 mg/kg favoring the appropriate dosing schedule.

The pharmacodynamic (PD) profile revealed a decrease in BCL-XL expression in peripheral WBCs, which was rapid after administration of DT2216. Furthermore, we observed that with a biweekly dose of DT2216 (0.4 mg/kg, BCL-XL expression continued to remain low while simultaneously not significantly affecting VHL expression levels and deemed the RP2D. A clinically relevant and safe concentration of DT2216 in plasma was achieved, demonstrating the degradation of BCL-XL and confirming the biological effect without partial or complete responses. Notably, in our preclinical studies, BCL-XL degradation in MOLT-4 T-ALL xenografts lasted much longer (approximately 2 weeks) than that in human peripheral WBCs after a single dosing, suggesting that the effect of DT2216 on tumor levels of BCL-XL might be underestimated based on its effect on WBC BCL-XL [5].

The advanced solid tumor population of patients in the study was heavily pre-treated with >3 previous lines of therapy and a significant disease burden. Although stable disease was the best overall radiographic response in 20% of patients given the early phase nature of the trial, we observed in our exploratory secondary analysis, a median overall survival of 7.9 months in a heavily pretreated population with a diverse disease spectrum. As shown previously in the preclinical and clinical studies by us and others, most solid tumors rarely depend only on BCL-xL for survival and thus respond poorly to the single agent treatment that targets BCL-xL alone except cutaneous T cell lymphoma (CTCL) [5, 7–10]. DT2216 was primarily designed to be combined with conventional chemotherapy or targeted therapy to overcome drug resistance. This is because these therapies can stimulate the expression of pro-apoptotic proteins via activation of the p53 pathway or inhibit the expression of other BCL-2 anti-apoptotic proteins (such as MCL-1 by paclitaxel) to make cancer cells more dependent on BCL-xL for survival [8, 11]. For example, preliminary studies have shown that the combination of DT2216 and paclitaxel is very effective in eradicating drug-resistant ovarian cancer cells [8]. This is in part because paclitaxel can inhibit MCL-1 transcription and translation via induction of mitotic arrest, which makes ovarian cancer cells highly sensitive to DT2216-induced BCL-xL degradation. In addition, our preclinical studies also showed that the combination of DT2216 with various targeted-therapeutic agents (such as BCL-2 inhibitors, mTOR inhibitors, and KRAS G12C inhibitors) as well as other conventional chemotherapeutic agents (such as gemcitabine, irinotecan, and azacytidine) can synergistically kill various cancer and leukemic cells [5, 10, 12–15]. Further studies with these different combinations can also expand the clinical application of DT2216. DT2216 will be tested in two recently initiated phase II studies: DT2216 plus paclitaxel to treat drug resistant ovarian cancer (NCT06964009) and DT2216 plus irinotecan to treat metastatic fibrolamellar hepatocellular carcinoma (NCT06620302).

Overall, limitations of the study do include the fact that this was an early phase clinical trial with a relatively small number of patients. Also, the patient population was heterogenous with several different tumor types with exposure to several different lines of standard of care therapies. These factors make interpretation of efficacy data limited. The use of surrogate tissue WBCs for PD analysis was another limitation of our study.

Currently, several other PROTACs are under clinical development, including degraders of Bruton tyrosine kinase (BTK), estrogen receptor (ER), Ikaros, and Aiolos transcription factors through Cereblon E3 (CRBN E3) ligase and KRAS G12C/D, but none has been approved by the US FDA for clinical use [16–19]. Another PROTAC targeting BCL-XL pending clinical evaluation utilizing CRBN E3 ligase, which is also not expressed in human platelets, would be an alternative therapeutic approach [20].

## Conclusion

DT2216 is safe and tolerable in patients with refractory advanced solid tumors, with one dose-limiting toxicity of asymptomatic grade 4 thrombocytopenia and no other safety signals. DT2216 0.4 mg/kg was determined to be RP2D.

## Data Availability

No datasets were generated or analysed during the current study.
