# Correction: First in human phase 1 study of DT2216, a selective BCL-xL degrader, in patients with relapsed/refractory solid malignancies

**DOI:** 10.1186/s13045-026-01837-z

**Published:** 2026-08-10

**Authors:** Daruka Mahadevan, Minal Barve, Devalingam Mahalingam, Jay Parekh, Michael Kurman, James Strauss, Larry Tremaine, Robert Hromas, Joshua Sills, John McCulloch, John Harkey, Stacy Suberg, Lisa Zimmerman, Guangrong Zheng, Daohong Zhou

**Affiliations:** 1 Mays Cancer Center, San Antonio, TX USA; 2 Minal Barve Sarah Cannon Cancer Research, Dallas, TX USA; 3 Devalingam Mahalingam Robert H. Lurie Comprehensive Cancer Center, Chicago, IL USA; 4 Jay Parekh Mays Cancer Center, San Antonio, TX USA; 5 Dialectic Therapeutics, Dallas, TX USA

**Correction: Journal of Hematology & Oncology (2025) 18:98**

10.1186/s13045-025-01753-8

The original article erroneously omits a Competing Interests statement which is listed ahead in this Correction article:

G. Zheng and D. Zhou are inventors of patent applications for the use of BCL-xL PROTACs (including DT2216) as antitumor agents. G. Zheng, D. Zhou, D. Hromas, J. Harkey are co-founders of and have equity in Dialectic Therapeutics. J. Strauss, L. Tremain, J. Sills, and J. McCulloch are employees of and have equity in Dialectic Therapeutics. M. Kurman, S Suberg, and L. Zimmerman are consultant of Dialectic Therapeutics. Dialectic Therapeutics is developing BCL-xL PROTACs (including DT2216) to treat cancer.

